# UNDERSTANDING RESOURCE PROFILES OF OLDER LEARNERS TO PROMOTE RECURRING ENGAGEMENT IN LEARNING

**DOI:** 10.1093/geroni/igad104.1531

**Published:** 2023-12-21

**Authors:** Pildoo Sung, Ad Maulod, Angelique Chan, Rahul Malhotra

**Affiliations:** Hong Kong Baptist University, Hong Kong, Hong Kong; Duke-NUS Medical School, Singapore, Singapore; Duke-NUS Medical School, Singapore, Singapore; Duke-NUS Medical School, Singapore, Singapore

## Abstract

Understanding characteristics of recurring learners among older adults is important for designing targeted strategies to promote lifelong learning. Studies have shown that older adults with human, social, and psychological resources are more likely to engage in learning. However, less is known about the interplay of these resources in motivating older learners to remain engaged in learning. Using longitudinal data from 515 older learners, aged 50 years and above, collected in Singapore in 2017-2018, we examined distinct resource profiles among older learners and their impact on recurring engagement in learning. Latent profile analysis explored (1) clusters of individuals with distinct resource profiles at baseline, comprising varying levels of human (education), social (civic attitudes, civic behaviors, and loneliness), and psychological resources (motivation to learn and control/autonomy/self-realization/pleasure), and (2) whether the resource profiles were associated with recurring engagement in learning (defined as attending another course within six months from baseline). Empirical analyses identified four resource profiles: (1) educated, resourceful (42.2% of older learners), (2) less educated, less resourceful (24.5%), (3) less educated, resourceful (20.0%), and (4) educated, less resourceful (17.0%). ‘Educated, less resourceful’ learners had a lower likelihood of recurring engagement in learning than ‘educated, resourceful’ learners. In summary, older adult learners are likely to possess varying levels of human, social, and psychological resources, which predict their recurring engagement in learning. Interventions helping older learners enrich their civic and psychological resources may facilitate their continued engagement in learning.

